# Comparison of medical versus surgical management of liver lobe torsion in rabbits

**DOI:** 10.18849/ve.v9i3.687

**Published:** 2024-07-19

**Authors:** Rachel Sibbald

**Affiliations:** Abercorn Vets, United Kingdom

**Keywords:** anaemia, anaesthesia, liver lobe torsion, medical, rabbit, surgery

## Abstract

**PICO question:**

Does medical management of liver lobe torsion in rabbits reduce mortality compared with surgical intervention?

**Clinical bottom line:**

**Category of research:**

Treatment.

**Number and type of study designs reviewed:**

Six studies were appraised in total. This consisted of three retrospective cohort studies and three case series.

**Strength of evidence:**

Weak.

**Outcomes reported:**

Medical management carries a similar 7-day survival rate compared to surgical treatment; however, long-term morbidity and mortality is increased compared with surgery. Delayed diagnosis and severe anaemia negatively affect outcomes of both groups.

**Conclusion:**

Medical management does not reduce mortality compared with surgical treatment and within the literature there appears to be a preference for surgery when treating rabbits with liver lobe torsion.

## How to apply this evidence in practice

The application of evidence into practice should take into account multiple factors, not limited to: individual clinical expertise, patient’s circumstances and owners’ values, country, location or clinic where you work, the individual case in front of you, the availability of therapies and resources.

Knowledge Summaries are a resource to help reinforce or inform decision-making. They do not override the responsibility or judgement of the practitioner to do what is best for the animal in their care.

## Clinical scenario

You are presented with a lethargic, anorexic rabbit which arrived as an emergency case after the owner noticed a sudden change in demeanour. On the clinical exam you notice the mucous membranes are pale and the cranial abdomen is firm and painful. Blood analysis reveals severe anaemia with raised liver enzymes. Ultrasonography confirms the suspicion of a liver lobe torsion with no doppler colour blood flow identified in a swollen lobe of liver surrounded by a small volume of free fluid. The owner wishes to pursue treatment but is very concerned about the risk of anaesthesia and surgery and wants to know if this can be avoided. An alternative consideration is medical management.

## The evidence

All of the studies identified are retrospective in nature and include cohorts and case series. Case series (Leonard et al., 2022; Stanke et al., 2011; Wenger et al., 2009) represent the weakest evidence available in this Knowledge Summary, as there is often an absence of data on potential confounding factors and there can be a range of bias that stem from issues such as loss to follow up or recall bias. In this Knowledge Summary, only descriptions of surgical treatment are reported and no evidence of case series surrounding medical management for comparison were found. This could represent a failure to diagnose the condition without exploratory surgery or bias in reporting surgical cases. Collectively the studies provide valuable information to aid clinicians with treatment of this rare condition, given the reported relative success of surgical intervention.

The cohort studies (Graham et al., 2014; Ozawa et al., 2022; Sheen et al., 2022) provide comparative data of medical and surgical treatment, allowing a more balanced evaluation in relation to the PICO question and a moderate strength of evidence when the limitations of studying this type of condition are considered. Both larger cohort studies (Ozawa et al., 2022; Sheen et al., 2022) found that the 7-day survival rate did not differ significantly between the medical and surgical groups, indicating medical management does not reduce mortality compared with surgery.

## Summary of the evidence

**Graham et al. (2014):** 

Population	Rabbits diagnosed with liver lobe torsion at Angell Animal Medical Centre (Boston, USA) between January 2007 and March 2012.
Sample Size	16 Rabbits.
Intervention Details	14/16 rabbits had a complete blood count performed. 12/16 had abdominal radiographs. 14/16 had abdominal ultrasonography performed which was diagnostic in all cases. 9 rabbits underwent exploratory surgery and liver lobectomy: Surgical correction involved circumferential ligature occlusion of the lobe/s and transection distal to the ligatures. Histologic examination was performed in all cases and was consistent with liver lobe torsion. 7 rabbits received supportive medical treatment only: Supportive care included administration of subcutaneous fluids, analgesia, antibiotics, supplemental feeding and prokinetic agents.
Study design	Retrospective single centre cohort study.
Outcome studied	Mortality rate of surgical and medical management.
Main findings (relevant to PICO question)	9/9 rabbits treated surgically survived. 3/7 rabbits treated medically survived. All 12/16 rabbits that survived to discharge are reported to have had no recurrent episodes of liver lobe torsion. 3/7 rabbits that survived and were treated medically reportedly had multiple episodes of recurrent gastrointestinal stasis that responded to medical management. The median hospital stay for medically treated rabbits was 2 days The median hospital stay for surgically treated rabbits was 4 days.
Limitations	2 rabbits within the medical treatment group that died had delayed diagnosis and concurrent illness but were still included in the outcome analysis. The first rabbit was not diagnosed until post-mortem. This animal also had toxic blood lead levels and decompensated despite chelation therapy. Necropsy confirmed a concurrent liver lobe torsion. The second rabbit was initially treated on an emergency basis however no diagnostic testing was performed until 2 days later. Surgical treatment was recommended but the rabbit died before surgery. Delay in diagnosis could have contributed towards the lower success rate in the medical treatment group It is unclear if the medical management regime was standardised within the medical group whilst the surgical technique appears relatively consistent. Details of medical treatment are lacking (i.e. specific drugs and dosages’). The paper does not detail the haematological and biochemical parameters of animals pertinent to their intervention group. General anaesthesia details from the surgical group are lacking. It is unclear if every case was offered equal opportunity for both medical or surgical treatment or what criteria was used to determine suitability for each treatment. As this is a retrospective, descriptive study, there is no control for chance, bias, and confounders.

Table note...

**Leonard et al. (2022):** 

Population	Rabbits diagnosed with a caudate liver lobe torsion at University of Wisconsin Veterinary Care (USA) between January 2018 and October 2021.
Sample Size	22 rabbits.
Intervention Details	21/22 rabbits had a biochemistry panel alongside measurement of packed cell volume and total solids. 6/22 were diagnosed via ultrasonography. 14/22 were diagnosed via abdominal computed tomography (CT). 2/22 were diagnosed via abdominal CT combined with ultrasonography. 22 rabbits underwent exploratory surgery and liver lobectomy: Surgical correction was performed using 2 techniques (13/22 via a ventral midline approach and 9/22 via a paracostal approach).
Study design	Retrospective single centre case series.
Outcome studied	Mortality rate of each surgical approach. Time to eating. Anaesthesia/surgical time.
Main findings (relevant to PICO question)	The mortality rate in the ventral midline surgical cases was 5/13 (38.5%). The mortality rate in the paracostal surgical cases was 0/9 (0%). Overall survival of both groups was 17/22 (77.2%). There was no statistical difference in anaesthetic or surgical time between the 2 groups. The median hospital stay for treated rabbits was 2 days.in both groups.
Limitations	It is not clear how long it was between diagnosis and surgical intervention for each rabbit. There was lack of control for anesthesia protocols, attending surgeon and anesthetist. It is not clear if rabbits had medical stabilisation performed prior to surgery and whether this was standardised between rabbits. General anaesthesia details are lacking as well as whether the anaesthetist was consistent in both groups. It is unclear what criteria was used to determine suitability for each surgical approach beyond surgeon preference and the study identified that this was because there was a reluctance to try a new surgical technique. As this is a retrospective, descriptive study, there is no control for chance, bias, and confounders. There are no medical cases for comparison in relation to the PICO question. It does not appear histopathology was performed in any surgical cases to confirm the diagnosis. Some rabbits received a blood transfusion but details surrounding indications for this are lacking.

Table note...

**Ozawa et al. (2022):** 

Population	Rabbits with liver lobe torsion at 4 veterinary referral hospitals (unknown, USA) between 1 January 2010 and 31 July 2020.
Sample Size	82 rabbits.
Intervention Details	9/82 (11%) rabbits were euthanised or died on presentation. 50/82 rabbits underwent surgical liver lobectomy: Surgical technique was described in 44 rabbits – suture ligation (26), instrument ligation (15), surgical looping device (2) and a vessel sealing device (1). Surgical complications included haemorrhage and incomplete resection. 23/82 rabbits received medical treatment only, however supportive care was provided to surgical rabbits too which included: Crystalloid fluid therapy with 1 rabbit also receiving hypertonic saline. Antibiotics were prescribed in 61/82 (74.4%) of rabbits. 67/82 (81.7%) rabbits received opioid analgesia including buprenorphine or a full mu agonist. 9/82 (11%) received a blood transfusion.
Study design	Retrospective multi-centre cohort study.
Outcome studied	Clinicopathological findings in rabbits with liver lobe torsions. Prognostic indicators. Mortality rate of surgical and medical management.
Main findings (relevant to PICO question)	36/50 (72%) of rabbits that underwent surgical intervention survived at least 7 days. 14/23 (60.9%) of medically treated rabbits survived at least 7 days. Overall, 32/82 (39%) rabbits died within 7 days of presentation and 50/82 (61%) survived. The 7-day survival rate did not differ significantly between the medical and surgical groups. The peri-anaesthetic mortality rate was 9/50 (17%). Median survival time following medical management was 530 days versus 1452 days with surgical treatment. Rabbits with caudate torsions were more likely to survive >7 days than those with right liver lobe torsions. A higher heart rate (HR) was associated with a worse outcome (P = 0.013; HR for each 10-beats/min increase in HR, 1.1; 95% CI, 1.02 to 1.19). For each additional day without defaecating, rabbits had a significantly lower survival time (P < 0.001; HR, 1.77; 95% CI, 1.3 to 2.41). Rabbits that received tramadol had longer survival time, compared with those that did not (P = 0.018; HR, 0.2; 95% CI, 0.06 to 0.7).
Limitations	The distribution of cases amongst the 4 hospitals has not been analysed and therefore is a source of potential bias if variation in treatment strategy and assessment/interpretation of results exists. 29/82 (35.4%) of rabbits were referred from another vet. This may create bias towards more severely affected rabbits being documented in the study. Statistical analysis of the biochemical and haematological parameters pertaining to each group would have been useful to identify any trends in data. The distribution of referral cases to each group has not been described. It is unclear if every case was offered equal opportunity for both medical or surgical treatment or what criteria was used to determine suitability for each treatment. There is no information surrounding concurrent co-morbidities which may have affected outcome in both groups. As this is a descriptive study, that is retrospect in nature, there was no control for chance, bias and confounders.

Table note...

**Sheen et al. (2022):** 

Population	Rabbits with liver lobe torsion torsion at Sydney Exotics and Rabbit Vets, Australia studied between 2016 and 2021.
Sample Size	40 rabbits.
Intervention Details	Packed cell volume and plasma biochemical testing was performed in 39/40 rabbits. Diagnostic imaging was performed in 38/40 rabbits. 15/40 (37.5%) of rabbits were treated medically, this included: Active warming if hypothermic. Opioid analgesia (buprenorphine 0.03 mg/kg subcutaneously (SC) or intravenously (IV) Q8h or 2 μg/kg/h fentanyl constant rate infusion). A dose range of 2–5 mg/kg ranitidine per os (PO) or IV Q12h or cisapride 0.5 mg/kg PO Q12h. Supplemental feeding with a herbivorous diet. Antibiotics were used to prevent bacterial translocation and included the use of procaine penicillin 30mg/kg Q24h SC and 20 mg/kg metronidazole Q12h (PO) or 7.5 mg/kg metronidazole Q12h IV. Antibiosis was continued for 2 weeks. 23/40 (57.5%) of rabbits were treated surgically in addition to the medical therapy described above. This included: General anaesthesia and surgical resection of the affected lobe. Ligation and occlusion of the vascular pedicle was performed using metal ligation clips, an electrosurgery bipolar vessel sealing system, surgical stapling devices, or distal transection. Rabbits with a packed cell volume (PCV) of less than 20%. (7/23) in the surgical group received a whole blood transfusion from a healthy donor.
Study design	Retrospective single centre cohort study.
Outcome studied	Subjective outcomes measured included return to ‘normal’ defaecation and food intake. Objective outcomes included improvement of PCV and biochemical parameters and survival 4 weeks after diagnosis.
Main findings (relevant to PICO question)	Overall, 30/40 (75%) of rabbits survived 15/15 (100%) of medically treated rabbits presented with reduced appetite. Of these, 12/15 (80%) survived 23/23 (100%) of rabbits that underwent surgery presented with reduced appetite. 19/23 (82.6%) of these rabbits Surgical intervention did not confer a significant difference in outcome compared to medical management (odds ratio 0.77, 95% confidence interval 0.15–10, P = 0.761). The peri-anaesthetic mortality rate was 4/23 (17.4%). For each percentage drop in PCV, the odds of a poor outcome increased by 12%. For each single unit increase (mmol/L) of urea, the odds of a poor outcome increased by 13%.
Limitations	Although a common inherent limitation of a retrospective case series is a lack of record keeping standardisation, it is unclear if every case was offered equal opportunity for both medical or surgical treatment or what criteria was used to determine suitability for each treatment. It is unclear why only the rabbits in the surgical group were offered a blood transfusion peri-operatively if PCV was less than 20%. This intervention is a potential confounding factor as this could have directly affected outcome/chance of survival. It is unclear if all animals were first opinion or referred from other vets (this could possibly result in bias towards more severely affected rabbits). 1 rabbit was grouped in both the surgical and medical group and included in statistical analysis despite ultimately receiving surgical treatment and surviving. 2 rabbits in the medical group had a delay in diagnosis. One rabbit was only diagnosed shortly before euthanasia and 1 on post-mortem examination. Both rabbits had a PCV < 20%. Imaging used for diagnosis was not standardised, with some rabbits undergoing CT scanning and some rabbits ultrasonographic diagnosis. There is no information surrounding concurrent co-morbidities or long-term medications which may have affected outcome in both groups. As this is a descriptive study that is retrospect in nature, there is no control for chance, bias, and confounders.

Table note...

**Stanke et al. (2021):** 

Population	Rabbits with liver lobe torsion evaluated at Angell Animal Medical Centre (Boston, USA) between June 2007 and March 2009.
Sample Size	4 rabbits.
Intervention Details	Case 1 - Exploratory laparotomy The rabbit was treated medically for 24 hours prior to surgery and was administered Lactated Ringer's Solution (4 ml/kg), intravenously (IV), Q1h and then 33 ml/kg (15 ml), subcutaneously (SC), Q8h), buprenorphine (0.04 mg/kg, SC, Q8h), enrofloxacin (10 mg/kg, per os (PO), Q12h), and metronidazole (20 mg/kg, PO, Q12h). Alongside syringe feeding with a herbivorous liquid diet (15 ml/kg, PO, Q8h). Premedication for surgery was achieved with buprenorphine 0.04 mg/kg and midazolam 0.5 mg/kg intramuscularly (IM) alongside meloxicam 0.4 mg/kg SC. Isoflurane with oxygen was delivered by an anaesthetic chamber followed by mask induction until the rabbit’s anaesthetic plane allowed endotracheal intubation. The affected liver lobe was occluded at the hilus with 2 circumferential ligatures of 2-0 polydioxanone suture and removed via transection distal to the ligatures. The medical treatment regimen was continued post-operatively and administration of metoclopramide hydrochloride (0.5 mg/kg, PO, Q8h) was commenced 2 days following surgery to increase gastrointestinal motility. Case 2 – Exploratory laparotomy The rabbit was treated medically for 24 hours prior to surgery and received Lactated Ringer's Solution (33 ml/kg, SC, Q8h), buprenorphine (0.04 mg/kg, SC, Q8h), and syringe feeding (15 ml/kg, PO, Q8h). The rabbit was premedicated with midazolam (0.7 mg/kg, IV) and butorphanol (0.2 mg/kg, IV). Anaesthesia was induced with isoflurane and oxygen in an anaesthetic chamber followed by mask induction until the rabbit’s anaesthetic plane allowed endotracheal intubation. Two circumferential ligatures of 3-0 polydioxanone suture were placed around the hilus of the torsed liver lobe, which was removed via transection distal to the ligatures. Following surgery, the rabbit was administered Lactated Ringer's Solution (4 ml/kg, IV, Q1h and then 33 ml/kg, SC, Q8h), buprenorphine (0.04 mg/kg, SC, Q8h), enrofloxacin (10 mg/kg, PO, Q12h), metoclopramide (0.5 mg/kg, PO, Q8h), and meloxicam (0.3 mg/kg, PO, Q12h). Alongside syringe feeding with a herbivorous liquid diet (15 ml/kg, PO, Q8h). Case 3 – Exploratory laparotomy The rabbit was admitted to the hospital and given Lactated Ringer's Solution (4 ml/kg, IV, Q1h) with concurrent heat support overnight. 24 hours later, the rabbit was premedicated with midazolam (0.3 mg/kg, IM), buprenorphine (0.04 mg/kg, IM), and glycopyrrolate (0.02 mg/kg, SC). Anaesthesia was induced with isoflurane and oxygen delivered by an anaesthetic chamber followed by mask induction until the rabbit’s anaesthetic plane allowed endotracheal intubation. Three circumferential ligatures of 3-0 polypropylene suture were placed around the hilus of the torsed caudate lobe, and 2 stainless steel ligation clips were placed distal to the ligatures and the lobe was transected between the clips. Following surgery, the rabbit was administered Lactated Ringer's Solution (4 ml/kg, IV, Q1h and then 33 ml/kg, SC, Q8h), buprenorphine (0.04 mg/kg, SC, Q8h), enrofloxacin (10 mg/kg, PO, Q12h), metronidazole (20 mg/kg, PO, Q12h), meloxicam (0.3 mg/kg, PO, Q12h) and cisapride (0.5 mg/kg, PO, Q8h), and syringe feeding (15 ml/kg, PO, Q8h). Case 4 – Exploratory laparotomy The rabbit was admitted to the hospital and administered Lactated Ringer's Solution (4 ml/kg, IV, Q1h and then 33 ml/kg, SC, Q8h), buprenorphine (0.04 mg/kg, SC, Q8h), enrofloxacin (10 mg/kg, PO, Q12h), metoclopramide (0.5 mg/kg, PO, Q8h), and meloxicam (0.3 mg/kg, PO, Q12h) and syringe feeding (15 ml/kg, PO, Q8h). The rabbit was premedicated with midazolam (0.5 mg/kg, SC), buprenorphine (0.04 mg/kg, SC), and glycopyrrolate (0.02 mg/kg, SC). Anaesthesia was induced with isoflurane and oxygen delivered by an anaesthetic chamber followed by mask induction until the rabbit’s anaesthetic plane allowed endotracheal. Three circumferential sutures of 3-0 nylon suture were placed around the hilus of the torsed caudate lobe, which was removed via transection distal to the sutures.
Study design	Retrospective case series.
Outcome studied	Surgical success (subjectively measured by wellness postoperatively, objectively measured by documentation of survival).
Main findings (relevant to PICO question)	All 4 rabbits survived surgery. Case 1 was still alive at 43 months post-surgery, case 2 was alive at 41 months post-surgery, case 3 was alive at 25 months post-surgery, and case 4 was alive 22 months after surgery. The mainstay of medical support to surgery included the administration of buprenorphine, meloxicam, lactated ringers solution (LRS), syringe feeding, antibiosis, and gastrointestinal prokinetics.
Limitations	There were no medically treated rabbits for comparison. The sample size is small and there is no quantitative/statistical data. As this is a descriptive study, that is retrospect in nature, there was no control for chance, bias, and confounders. Details of inclusion criteria are missing, there is therefore a risk of selection bias in this report.

Table note...

**Wenger et al. (2009):** 

Population	Rabbits with liver lobe torsion.
Sample Size	3 rabbits.
Intervention Details	Haematology was analysed for cases 1 and 2, and mean packed cell volume (PCV) was 16.6%. Biochemical parameters showed marked increases in liver enzymes (alanine transaminase and aspartate aminotransferase) in all 3 rabbits. Case 1 (5 kg) – Surgical case although initially managed medically for 24 hours: Hartmann's solution was delivered at 30 ml/hr intravenously. 10 ml/kg oral electrolyte solution. 0.4 mg/kg buprenorphine intravenously (IV). 2 mg/kg meloxicam once daily subcutaneously. 5 mg/kg ranitidine per os (PO) twice daily (BID). Fine needle aspiration of the liver revealed normal cytology. The rabbit was premedicated with 0.06 mg/kg of fentanyl and 2 mg/kg of fluanisone intramuscularly (IM), followed by 8 mg/kg propofol and 3 mg/kg of midazolam intravenously. Anaesthesia was maintained with isoflurane in oxygen via an endotracheal tube. The affected liver lobe was resected by clamping the pedicle and ligating at the site of the torsion with absorbable suture. The abdominal incision was closed in three layers with absorbable suture in a simple continuous pattern. Case 2 (2.2 kg) – Medical management: Saline was given IV at 12 ml/hour. 2 ml high fibre paste was administered orally 3 times daily (TID). 0.03 mg/kg buprenorphine was given IV TID. 36 mg/kg meloxicam was given PO once daily (SID). 4 mg/kg ranitidine was administered PO BID. 13 mg/kg trimethoprim-sulfamethoxazole was given PO BID. Fine needle aspiration of the liver revealed normal cytology. Case 3 (2.6 kg) – Medical management resulting in euthanasia (owner declined exploratory laparotomy): Hartmann's solution was given IV at 12 ml/hour. 0.02 mg/kg buprenorphine intravenously (IV).
Study design	Retrospective case series.
Outcome studied	Confirmation of diagnosis (histopathology on postmortem). Mortality.
Main findings (relevant to PICO question)	No rabbits survived. **Case 1 **died during recovery from surgery. The resected liver was sent for histopathology and was consistent with a liver lobe torsion. **Case 2** died 2 days after initial presentation following medical management. A post-mortem was performed and histopathology was consistent with a liver lobe torsion. **Case 3** was euthanised following a very short period of medical treatment. On post-mortem 50 ml of serosanguinous fluid was found in the abdomen, and histopathology was consistent with a liver lobe torsion.
Limitations	Interventions differed between patients and data shared was reliant upon accurate record keeping/reporting. 1 rabbit was euthanised before any type of treatment commenced, reducing the already small sample size. The significance between the intervention and the outcome is unclear as no rabbits survived. As this is a descriptive study, that is retrospect in nature there is no control for chance, bias and confounders. There are no quantitative/statistical data or calculations, so there is a risk of over-interpretation.

Table note...

## Appraisal, application and reflection

Liver lobe torsion (LLT) is a rare condition described in many species including humans, dogs, pigs, rats, and rabbits (Graham and Basseches, 2014). The aetiology is unknown but predisposing factors in humans and dogs are thought to include congenital absence of hepatic ligaments, dilation of abdominal organs, and surgical or external trauma to the abdomen (Graham and Basseches, 2014). Acute venous infarction, thrombus formation, and hepatic necrosis can result in the development of effusion of the abdomen and thorax, haemoabdomen, and ultimately, acute death. Disseminated intravascular coagulation has been reported as a complication of bacterial toxin and ischaemic by-product release. In dogs, the left lobe is most affected; however, in rabbits the caudate lobe located in the right cranial abdomen is more commonly involved (Oglesbee and Lord, 2020). This is thought to be because of the narrow attachment to the dorsal hilar region of the liver (Summa and Brandão, 2017). This would suggest when applying this knowledge to clinical practice that further investigation is warranted when abdominal pain or mass effect is palpated in the right cranial abdomen of rabbits.

Clinical signs are non-specific and can mimic other disorders causing pain. The largest cohort study (Ozawa et al., 2022) found that the most common presenting complaint was reduced appetite or anorexia (76/82 (92.7%)), followed by lethargy (46/82 (56%)) and decreased defaecation (38/82 (46.3%)) of rabbits. In clinical practice, this is a very common presentation and highlights the need for thorough clinical examination and investigation into what are vague and non-pathognomonic symptoms. Clinical examination may reveal abdominal pain, dehydration, dull mentation, pale mucous membranes, and decreased borborygmi (Graham and Basseches, 2014).

Diagnosis is achieved through a combination of evaluation of clinical signs, blood testing and diagnostic imaging. Sheen et al. (2022) found that anaemia and elevated plasma alanine aminotransferase and blood urea nitrogen were common clinicopathologic results. This correlates to the findings by Ozawa et al., (2022) where 47/82 (59.5%) of rabbits were anaemic. Ozawa et al., (2022) also reports that rabbits with moderate or severe anaemia were significantly less likely to survive for seven days (P = 0.006; OR, 4.41; 95% CI, 1.55–12.51) than rabbits with mild or no anaemia. In the study by Sheen et al., (2022) an identified limitation was a potential confounding factor within the surgical group. These rabbits were administered a blood transfusion from a healthy donor if the packed cell volume (PCV) was less than 20%, whereas the medical group was not offered this intervention. Of these rabbits, 6/7 (85%) survived, whilst non-survivors of the medical group all had a PCV of less than 20%. When evaluating the studies collectively, the use of blood transfusion was surprisingly uncommon (or not described) despite descriptions of rabbits demonstrating criteria that would normally necessitate transfusion. This includes a PCV of less than 20%, blood loss associated with collapse, ongoing haemorrhage or poor response to conventional shock therapy with crystalloid or colloid fluids (Lichtenberger, 2004). Identification of criteria for blood transfusion, using objective parameters such as a PCV, may help shape recommended treatment interventions in future studies of LLT. Diagnostic modality appeared to vary between institutions and within institutions for different cases. The large cohort study by Ozawa et al., (2022) used ultrasonography to diagnose all 82 cases whereas Sheen et al., (2022) and Leonard et al., (2022) relied on a combination of ultrasound and computed tomography (CT). In practice this may be limited by availability of equipment and user competency and anecdotally, ultrasonographic diagnosis may be hindered by gastrointestinal gas and fast respiratory motion artefact. A study by Specchi and d’Anjou (2019) concluded that either ultrasound or CT can be used independently to diagnose vascular disease including organ torsion in small animals but given the complexity of vascular anatomy their combined use likely yields higher diagnostic accuracy.

A common theme throughout the evaluation was lack of information surrounding the rationale for either surgical or medical management. This information is invaluable for clinicians in practice where lack of experience may hinder decision making. Trends of data would have been useful in the cohort studies to assess whether there were clinical parameters which influenced treatment modality. Equally, factors outside the animals’ clinical condition may have influenced treatment (such as owner consent), therefore, documentation of this would have been useful where available. None of the case reports or series included documentation of medical management, which could represent publication bias towards cases of positive surgical intervention. On balance, there is a strong recommendation in other species that surgical resection is necessary to avoid the effects of venous obstruction, such as thrombosis and necrosis (Swann and Brown, 2001). Despite the lack of comparison in relation to the PICO question the surgical case reports do provide value, as knowledge and experience can be transferred to readers and detail of anaesthesia protocols in critically ill rabbits may help clinicians in practice.

The studies which did involve medical management cases for comparison (Graham et al., 2014; Ozawa et al., 2022; Sheen et al., 2022; Wenger et al., 2009) varied somewhat in strategy; however, the principles (which are applicable to many conditions in rabbits) were essentially the same. Fluid therapy was delivered either intravenously or subcutaneously, a range of gastrointestinal prokinetics were used, opioids and non-steroidal anti-inflammatories were used as analgesia, and antibiotic use was common. Retrospective studies can lack control for chance, bias, and confounders, and as the interventions differed between patients, data shared was reliant upon accurate record keeping and reporting. In the Graham et al. (2014) study, two rabbits within the medical treatment group that died, had delayed diagnosis and concurrent illness but were still included in the outcome analysis. One of these rabbits had severe co-morbidities and therefore, data interpretation in this study should be approached with caution due to the small sample size (16 rabbits) and the inclusion of these animals. Sheen et al. (2022) categorised a rabbit in both the medical and surgical group despite it ultimately receiving surgery and surviving and in the same study two rabbits in the medical group had a delay in diagnosis. The same study showed no significant outcome difference between medical and surgical management. The authors suggest that prompt diagnosis within one day of presentation and evidence of relatively modest blood loss appeared to support successful medical management. Ozawa et al. (2022) also found patients that underwent surgery did not have a significantly different 7-day outcome, but median survival time was 530 days for rabbits that received only medical treatment and was 1452 days for rabbits that underwent surgery.

Therefore, despite the limitations identified within the retrospective studies examined (Graham et al., 2014; Ozawa et al., 2022; Sheen et al., 2022; Wenger et al., 2009), they do have significant value that allows clinicians to consider both medical and surgical options in the rabbit. Clinically, there is broad similarity between all the studies and the techniques used for medical support and this should provide a good starting point for supporting rabbit patients. However, further work is needed to evaluate the role blood transfusion has to play in improving patient outcomes in this condition, anaesthesia of surgical patients and a review of the methodology clinicians use when deciding whether to approach medical or surgical treatment would be beneficial.

Only one of the studies (Leonard et al., 2022) described a difference in surgical approach and made direct comparison of two techniques. The study described prior use of cadavers to assess feasibility of a paracostal approach to access the liver versus the more familiar ventral midline approach. Interestingly, the mortality rate of rabbits that underwent surgery via the paracostal approach was 0% compared to the 5/13 (38.5%) seen in the ventral midline group. The authors of this study proposed this may be because of the position of the rabbit during surgery. Anecdotally, more anaesthetic challenges are seen when rabbits are positioned in dorsal recumbency due to the gastrointestinal tract placing pressure on the vena cava resulting in trouble managing patient blood pressure and secondly a full stomach may impede respiratory movements if there is pressure on the diaphragm (Heskin, 2019). A third consideration is access during surgery and the authors found that there was improved exposure of the caudate lobes vascular pedicle when using the paracostal approach. This may explain the higher incidence of intra-operative haemorrhage in the ventral midline group due to obstructed access/vision. Further studies are required using larger case numbers to assess viability and practicality of the paracostal approach to determine whether this approach should be used preferentially to the midline approach if surgery is undertaken.

## Methodology

**Search strategy:** 

Databases searched and dates covered	CAB Abstracts on Ovid Platform from 1973 to 1 March 2024 PubMed accessed via NCBI interface from 1950 to 1 March 2024
Search terms	CAB Abstracts (Pet rabbit* OR domestic rabbit* OR rabbit OR rabbits OR Oryctolagus cuniculus) AND (liver lobe) PubMed (Pet rabbit* OR domestic rabbit* OR rabbit OR rabbits OR Oryctolagus cuniculus) AND (liver lobe torsion)
Dates searches performed:	01 Mar 2024

Table note placeholder

**Exclusion / inclusion criteria:** 

Exclusion	Irrelevant to PICO. Cadaveric study. Not available in English. Review article. Single case study.
Inclusion	Relevant to PICO question, peer-reviewed and available in English.

Table note placeholder

**Search outcome:** 

Database	Number of results	Excluded – Not relevant to PICO	Excluded – Cadeveric study	Excluded – Not available in English	Excluded – Not available online	Excluded – Review Article	Excluded – Single case study	Total relevant papers
CAB Abstracts	25	10	1	1	1	2	4	6
PubMed	59	5	2	0	1	4	0	4
Total relevant papers when duplicates removed								6

Table note placeholder

## ORCiD

Rachel Sibbald: https://orcid.org/0000-0003-1741-2434

## Conflict of interest

The author declares no conflicts of interest.

## Disclaimer

Knowledge Summaries are a peer-reviewed article type which aims to answer a clinical question based on the best available current evidence. It does not override the responsibility of the practitioner. Informed decisions should be made by considering such factors as individual clinical expertise and judgement along with patient’s circumstances and owners’ values. Knowledge Summaries are a resource to help inform and any opinions expressed within the Knowledge Summaries are the author’s own and do not necessarily reflect the view of the RCVS Knowledge. Authors are responsible for the accuracy of the content. While the Editor and Publisher believe that all content herein are in accord with current recommendations and practice at the time of publication, they accept no legal responsibility for any errors or omissions, and make no warranty, express or implied, with respect to material contained within. For further information please refer to our Terms of Use.
